# Subcutaneous Administration of Bortezomib: A Pilot Survey of Oncology Nurses

**DOI:** 10.6004/jadpro.2015.6.4.2

**Published:** 2015-07-01

**Authors:** Jasmine R. Martin, Nancy L. Beegle, Yanyan Zhu, Ellen M. Hanisch

**Affiliations:** 1 Millennium Pharmaceuticals, Inc., Littleton, Colorado;; 2 Cancer Clinics of Excellence, Greenwood Village, Colorado;; 3 Millennium Pharmaceuticals, Inc., a wholly owned subsidiary of Takeda Pharmaceutical Company Limited, Cambridge, Massachusetts;; 4 Hematology Oncology Northwest, Tacoma, Washington

## Abstract

Subcutaneous (SC) administration of the proteasome inhibitor bortezomib was approved in the United States and European Union in 2012. There is limited guidance regarding how to administer SC bortezomib and a general lack of clear direction on optimal techniques for administering SC chemotherapy injections. Nurses may be utilizing different techniques, and inconsistent techniques may result in injection-site reactions, causing patient discomfort and treatment cessatioin. This observational survey of oncology nurses in community oncology clinics aimed to identify techniques being used and explore nurses’ opinions about SC bortezomib administration. A 44-question electronic survey was developed, based on the current literature regarding appropriate techniques for administering SC injections. A total of 43 nurses from 17 clinics in 12 states responded. The majority (74%) had been practicing oncology nursing for at more than 5 years. Respondents predominantly used and preferred the abdomen for injections (88%); 81% used a skin lift to ensure injection into adipose tissue. There was no relationship between the angle of insertion and the needle length; 51% used an air-bubble technique. Nurses took 3–5 (49%), 5–10 (35%), 10–30 (9%), or > 30 (7%) seconds to administer each mL of SC bortezomib injection. All nurses completely/somewhat agreed that practice guidelines would be important for standardizing SC bortezomib administration. Advanced practice registered nurses (APRNs) shared the responsibility for ordering SC bortezomib, according to 53% of respondents. These findings could help APRNs improve the quality of patient care, may help minimize adverse events and maximize effective therapy, and could help inform the development of practice guidelines.

Multiple myeloma (MM) is the second most common hematologic malignancy in the United States (Siegel, Miller, & Jemal, 2015) and worldwide (Jemal et al., 2011), with an estimated 26,850 new cases and 11,240 deaths in the United States in 2015 (Siegel et al., 2015). The proteasome inhibitor bortezomib (Velcade), alone and in combination, is highly effective in the treatment of patients with MM, producing high response rates and resulting in improved overall survival across disease settings (Driscoll, Burris, & Annunziata, 2012; Jakubowiak, 2012; Moreau et al., 2012; San Miguel et al., 2013; Sonneveld et al., 2013). Grade ≥ 3 adverse events reported in ≥ 10% of patients in phase III clinical studies of single-agent intravenous (IV) bortezomib include peripheral neuropathy, thrombocytopenia, neutropenia, and anemia (Moreau et al., 2011; Orlowski et al., 2007; Richardson et al., 2005).

Bortezomib is approved for the treatment of MM in the United States (Millennium Pharmaceuticals Inc., 2014) and the European Union (Janssen-Cilag International NV, 2015). Bortezomib was initially approved in 2003 for administration by the IV route only; however, subcutaneous (SC) administration of bortezomib was approved in the United States in January 2012 (Millennium Pharmaceuticals Inc., 2014) and the European Union in September 2012 (Janssen-Cilag International NV, 2015), in addition to the IV route.

Approval was based on the findings of a phase III randomized study of SC vs. IV bortezomib in patients with relapsed/refractory MM, which demonstrated noninferior efficacy in terms of overall response rate after four cycles of single-agent treatment (Arnulf et al., 2012; Moreau et al., 2011). In addition, SC bortezomib resulted in fewer grade ≥ 3 adverse events than the IV route (57% vs 70%), less peripheral neuropathy of any grade (38% vs 53%, *p* = .044; Moreau et al., 2011), and fewer dose reductions due to adverse events (31% vs 43%; Moreau et al., 2011). The implications of dose reduction and discontinuation of therapy due adverse events can include treatment failure or less effective alternative therapy (McEwan et al., 2010).

There is limited guidance in the US Prescribing Information (PI; Millennium Pharmaceuticals Inc., 2014) and European Union Summary of Product Characteristics (Janssen-Cilag International NV, 2015) regarding how to administer SC bortezomib. The US PI states that bortezomib may be administered SC at a concentration of 2.5 mg/mL; it also notes that sites for each injection (thigh or abdomen) should be rotated and that new injections should be given at least one inch from an old site and never into areas where the site is tender, bruised, erythematous, or indurated (Millennium Pharmaceuticals Inc., 2014). The European Union Summary of Product Characteristics states that bortezomib is administered SC (at 2.5 mg/mL) in the thigh (right or left) or abdomen (right or left), that the solution should be injected SC at a 45°–90° angle, and that injection sites should be rotated for successive injections (Janssen-Cilag International NV, 2015). Both the US PI and the European Union Summary of Product Characteristics indicate that a less-concentrated bortezomib solution may be used if local injection-site reactions occur. The phase III study did not evaluate SC administration into the arm, and pharmacokinetic data from administration into the abdomen and thigh cannot be extrapolated to this site of administration.

More broadly, there is a lack of clear direction in the oncology literature on the optimal techniques for administering SC chemotherapy injections (Annersten & Willman, 2005). One systematic literature review identified inconsistencies in the information available, thus limiting the development of recommendations for optimal SC injection, and recommended the identification of how nurses actually administer SC injections (Annersten & Willman, 2005).

Studies in non-oncology fields have addressed the use of different needle sizes and lengths for SC injection (Akkus, Oguz, Uzunlulu, & Kizilgul, 2012; Arendt-Nielsen, Egekvist, & Bjerring, 2006; Birkebaek et al., 2008; Frid et al., 2010; Gibney, Arce, Byron, & Hirsch, 2010; Gill & Prausnitz, 2007; Hunter, 2008; McConnell, 1990; Pope, 2002; Rushing, 2004; Hansen & Matytsina, 2011), the use of dry needles and the air-bubble technique (Agac & Gunes, 2011; Frid et al., 2010; Hunter, 2008; Klingman, 2000; Kurtin, Knop, & Milliron, 2012; Lamblet, Meira, Torres, Ferreira, & Martucchi, 2011; Moore et al., 2007; Wooldridge & Jackson, 1988), the angle of insertion (Akkus et al., 2012; Gibney et al., 2010), and the duration of the SC injection (Balci Akpinar & Celebioglu, 2008; Zaybak & Khorshid, 2008).

Overall, the findings of these studies suggest that injection-site reactions, pain, and bruising can be reduced by using short needles, injecting at 45° into skin folds, using the air-bubble rather than the purge technique, and slower injections. Inconsistent or poor administration techniques may increase injection-site reactions and could contribute to patients stopping effective treatment, whereas good techniques can reduce these adverse events (Girouard & Theoret, 2008; McEwan et al., 2010). Advanced practice registered nurses (APRNs) can influence outcomes that are meaningful to patients by modeling the importance of consistent caring practice to patients and supporting professional collaboration with evidence-based practice (Gawlinski et al., 2013).

Therefore, it was important to identify how oncology nurses are administering SC bortezomib. This observational survey of oncology nurses practicing in community oncology clinics aimed to identify the techniques being used and to explore nurses’ opinions about the SC route of bortezomib administration.

## METHODS AND MATERIALS

**Survey Methodology**

A 44-question electronic survey on SC bortezomib administration was developed, with all questions based on the current literature regarding appropriate techniques for administering SC injections. Completion of the survey constituted consent to participate, and the New England Institutional Review Board granted exemption from review for the survey.

The questions explored the sites of administration, needle length, angle of needle insertion, use of the air purge or the bubble technique, and duration of administration. Questions were also included regarding the convenience of SC administration, nurses’ qualitative opinions on patients’ preferences for the administration site, correlation between facility layout and administration site, and nurses’ qualitative opinions about practice guidelines for SC administration. The validity of the content of the survey was confirmed through a review of the questions by five oncology nurses, one medical oncologist, and three experts in health economics and outcomes research.

**Participants**

Nurses at 19 community-based oncology clinics who had administered SC bortezomib were invited to participate, with limitations imposed on respondent numbers to ensure country-wide input. Responses per clinic were included based on the order in which they were received.

**Statistical Analyses**

All data were summarized using descriptive statistics. Proportion differences between groups were compared using Chi-square tests or Fisher’s exact tests (*p* < .05 regarded as statistically significant).

## RESULTS

**Survey Participants**

A total of 43 nurses from 17 clinics located in 12 states (Arkansas, California, Connecticut, Florida, Georgia, Illinois, Maryland, Massachusetts, Montana, Oklahoma, Pennsylvania, and Washington) responded (see Table). The majority (74%) had been practicing oncology nursing for more than 5 years, including 21% for more than 20 years. The Table summarizes respondents’ experience and responsibilities with regard to SC bortezomib administration. Most respondents (93%) indicated that oncologists were responsible for ordering bortezomib for SC administration; however, APRNs shared this responsibility, according to 53% of respondents. Overall, 26 nurses (60%) indicated that they always/sometimes provided input into the decision to use SC administration, with 40% always/sometimes responsible for bortezomib reconstitution.

**Table 1:**
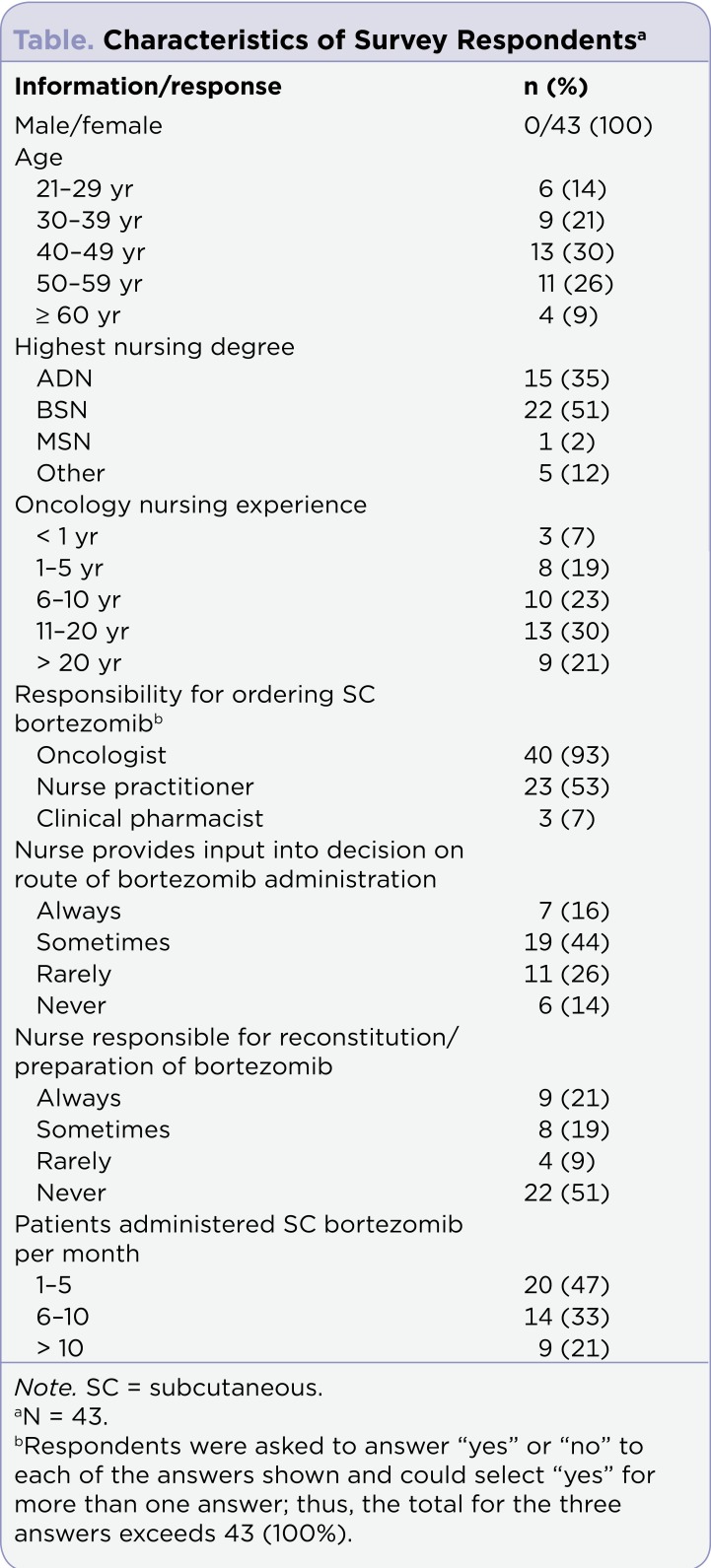
Characteristics of Survey Respondents^a^

**Injection Site**

**Figure 1:**
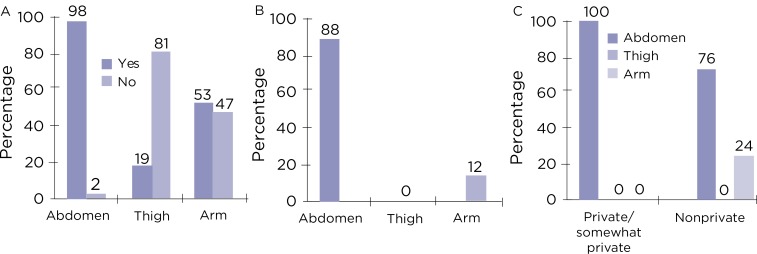
Oncology nurse respondents’ (A) injection-site selection, (B) injection-site preference, and (C) correlation between injection-site preference and facility layout, for administration of subcutaneous bortezomib (*p* = .02, based on Fisher’s exact test).

Nurses were asked to report which anatomic sites they used to administer SC bortezomib and which sites they preferred (Figure 1): 98%, 19%, and 53% reported using the abdomen, thigh, and arm, respectively (Figure 1A). The abdomen (88%) and arm (12%) were preferred (Figure 1B), primarily due to less irritation/pain (n = 17, 40%), the presence of more tissue/larger area (n = 15, 35%), ease of access (n = 11, 26%), and patient preference (n = 4, 9%).

Regarding patients’ preferences for injection site: 31 nurses (72%) believed that patients preferred receiving injections in the abdomen, and 12 nurses (28%) believed patients preferred injections in the arm. Eighteen nurses (42%) felt that privacy concerns for patients influenced SC bortezomib injection-site selection; six respondents (14%) indicated they did not think privacy was important to their patients when selecting injection sites.

Nurses’ injection-site preferences differed significantly according to the facility layout: all respondents indicated a preference for abdominal injection in private/somewhat private facilities, whereas the abdomen (76%) and arm (24%) were preferred in nonprivate facilities (*p* = .02; Figure 1C). The thigh was never a preferred site for SC bortezomib administration.

The most common reason given (n = 21) for preferring the abdominal site was reduction of local site irritation. Fourteen respondents (33%) felt that accessibility of the site impacted patient preference for the injection site, suggesting the rationale for preferring the arm; eight respondents (19%) noted that anatomic considerations impacted patient site preference, citing the amount of surface area and SC tissue; and three respondents (7%) did not give patients a choice of injection site other than the abdomen.

Subcutaneous injections were primarily given in an open infusion suite with curtains around each chair (n = 16, 37%); in an open infusion suite with chairs (n = 19, 44%); in a private examination room (n = 3, 7%); at a nurses’ station (n = 1, 2%); or in another location (n = 4, 9%). Twelve respondents (28%) thought that patients were concerned about exposure while receiving the SC injection, and 15 respondents (35%) noted that they could provide a private area in which to give the injection.

A total of 29 nurses (67%) rotated injection sites within the same anatomic area, 8 nurses (19%) rotated to different anatomic sites, and 6 nurses (14%) rotated at their discretion, with no designated pattern. All respondents documented the sites of SC bortezomib injection, and 10 nurses (23%) had an anatomic map in the patient’s chart to guide site rotation.

**Administration Technique**

**Figure 2:**
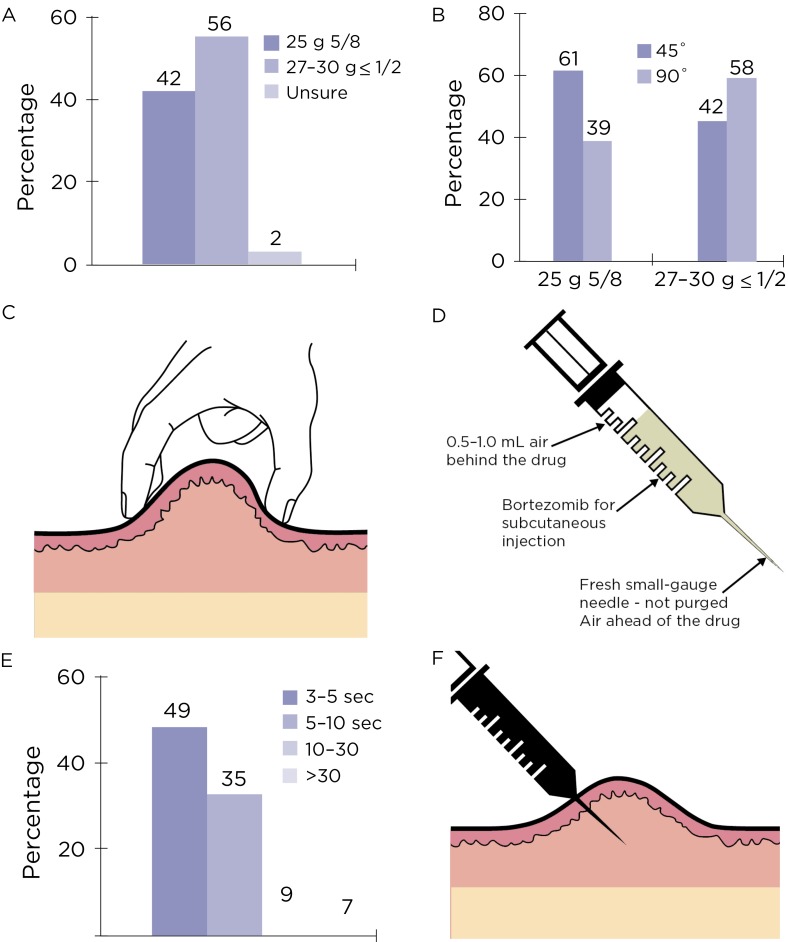
Responses from oncology nurses regarding (A) needle gauge and length and (B) needle size and angle of insertion (*p* = .21, based on Chi-square test). Also shown are (C) skin-lift technique, (D) airbubble technique, (E) responses from oncology nurses regarding time taken to administer each mL of subcutaneous bortezomib, and (F) 45° angle of insertion with skin lift (recommended for needles longer than 6 mm).

*Needle Size, Angle of Insertion, and Skin Lift*: Nurses were asked to report the needle size and injection technique that they used for SC bortezomib administration. Nurses used 25-gauge 5/8 inch (42%) and 27–30 gauge ≤ 1/2 inch (56%) needles (Figure 2A), with both 45° (61%, 42%) and 90° (39%, 58%) angles of insertion used with these respective needle sizes (*p* = .21; Figure 2B). Overall, 22 nurses (51%) used a 45° angle of insertion, and 21 nurses (49%) used a 90° angle. Respondents primarily used a skin lift (Figure 2C) to ensure injection into adipose tissue (n = 35, 81%).

*Needle Change, Air Bubble, or Needle Purge*: The majority of respondents (n = 40, 93%) routinely put a new needle on the syringe before administering the injection The air-purge technique was used by 49% of nurses (n = 21), with 51% (n = 22) using an air bubble (Figure 2D).

The most common reason given for use of the air-bubble technique was that nurses felt that what they were doing ensured the full dose was delivered and/or stayed in the SC tissue. Eleven respondents (26%) also noted that the air-bubble method decreased local-site irritation by either clearing the medication out of the needle or preventing medication from leaking back out onto the skin. The reasons cited for using the air-purge technique were to avoid injecting air into the patient and because this is the way they always give/were taught to give injections.

*Administration Time*: Nurses took various lengths of time to administer each mL of SC bortezomib injection (Figure 2E): 21 (49%), 15 (35%), 4 (9%), and 3 (7%) nurses, respectively, took 3–5, 5–10, 10–30, or > 30 seconds.

*Administration Guidelines*: Participants agreed that practice guidelines are important for consistency and quality care. The majority of nurses completely agreed (n = 17, 40%) or somewhat agreed (n = 24, 56%) that all nurses in their clinic used the same technique to administer SC bortezomib. Two nurses (5%) somewhat disagreed with this statement. All but one of the respondents completely agreed (n = 24, 56%) or somewhat agreed (n = 18, 42%) that it was important to patients for all nurses to be using the same administration technique for SC bortezomib. With regard to whether this was important to physicians, 21 (49%) completely agreed, 15 (35%) somewhat agreed, 5 (12%) somewhat disagreed, and 2 (5%) disagreed completely.

All nurses completely agreed (n = 33, 77%) or somewhat agreed (n = 10, 23%) that developing a practice guideline would be important for standardizing how and where SC bortezomib was administered. All but one respondent completely agreed (n = 29, 67%) or somewhat agreed (n = 13, 30%) that nurses would change their practice to be consistent with such a guideline.

Overall, 22 respondents (51%) indicated that there was a standard guideline in their clinic for SC administration of bortezomib; 9 (21%) indicated that there was not a standard guideline, and 12 (30%) were unsure. There was no association/correlation between whether a clinic had a standard guideline and the time to administer an injection (*p* = .19), use of the air-bubble technique (*p* = .31), or angle of insertion (*p* = .57).

From the qualitative responses, the most common explanations given for following practice guidelines centered on a desire to use evidence-based practice and to follow published guidelines where available. Five respondents (12%) indicated that they would change their behavior only if they saw patient benefit from the change. Clinic guidelines were also identified as important to nurses’ behavior, and eight respondents (19%) indicated that they would change their behavior only if their clinic approved the change. One respondent (2%) indicated that change in behavior depended upon what other practices were doing, and another commented that their method was correct, as evidenced by her own experience of minimal injection-site reactions.

*Convenience*: All but one nurse (98%) indicated that SC administration was much more or somewhat more convenient than IV administration, with 41 respondents (95%) indicating that SC administration took much less or somewhat less time than IV administration. Most nurses (n = 37, 86%) believed that, among those who had received both IV and SC bortezomib, patients preferred the SC route for reasons including less time spent at the clinic (n = 26), avoidance of IV access (n = 11), and less toxicity (n = 6). Only two nurses (5%) believed patients preferred IV administration, with four (9%) indicating patients had no preference between IV and SC administration.

Among qualitative answers regarding preference for SC administration, the most common reasons centered on the time saved due to not having to start an IV line, administer hydration, and administer premedications. Eight respondents (19%) considered the patient’s perspective in their answers, citing reduced stress (associated with obtaining IV access), less time required at the clinic, better patient compliance, and patient preference. Notably, seven respondents (16%) implied that they discussed the possibility of reduced side effects with their patients through use of SC administration, which was thought to influence patient preference for this route. One respondent (2%) noted that some patients reverted to IV administration secondary to skin irritations with SC injection.

## DISCUSSION

The findings from this survey of oncology nurses indicate the heterogeneity of techniques used for SC bortezomib administration. All the questions in the survey were based on the current literature regarding appropriate techniques for administering SC injections. However, inconsistency in the literature, lack of information in clinical trials on how SC chemotherapy is administered, and use of techniques based on tradition may prevent nurses from using best evidence for SC injections. Clinical studies specific to SC injections have demonstrated that some techniques result in reduced injection-site pain and bruising, improving patient satisfaction and adherence to treatment (Balci Akpinar & Celebioglu, 2008; Frid et al., 2010; Gibney et al., 2010; Gill & Prausnitz, 2007; Girouard & Theoret, 2008; Lamblet et al., 2011; Moore et al., 2007; Wooldridge & Jackson, 1988; Zaybak & Khorshid, 2008).

Therefore, it was important to describe how nurses are administering SC bortezomib. The experience of the oncology nurses who responded to the survey was substantial: More than half of respondents had been practicing oncology nursing for > 10 years, and more than half of respondents administered SC bortezomib to more than five patients per month. Therefore, the findings of this survey are highly relevant based on the respondents’ experience of administering SC bortezomib.

With regard to injection site, this survey indicates some discrepancies with the limited guidance on SC bortezomib administration provided in the US PI (Millennium Pharmaceuticals Inc., 2014), which lists only the thigh and abdomen as sites for injection, consistent with the sites used in the phase III study of SC vs. IV bortezomib (Arnulf et al., 2012; Moreau et al., 2011).

For example, the respondents primarily used and preferred the abdomen for SC bortezomib administration; additionally, the arm was frequently used, mainly due to convenience of access and patient preference for privacy, and the thigh was never preferred. Of interest in this regard, a retrospective analysis of injection-site reactions in 15 Japanese patients receiving SC bortezomib showed that grade 2 reactions were more common following administration in the thigh vs. the abdomen (9.2% vs. 1.1%, *p* = .014; Kamimura et al., 2013). This finding accords with the findings of this survey, in which the abdominal site was preferred due to reduction in local-site irritation.

The survey highlighted different practices with respect to needle length, skin lift, and angle of insertion. Studies have demonstrated that short needles are safer and better tolerated than longer needles for SC injections (Gibney et al., 2010). Skin thickness does not vary significantly in adults, whereas SC adipose tissue does vary in different anatomic sites, between genders, and with increased body mass index and waist circumference (Akkus et al., 2012; Gibney et al., 2010). Despite adipose tissue differences, small-gauge short needles (4–6 mm) have been shown to deliver SC medications effectively, even in obese patients, whereas longer needles (8–12 mm) may result in intramuscular (IM) injection (Akkus et al., 2012; Frid et al., 2010; Gibney et al., 2010).

In a study of 388 adults with diabetes, the use of short (5 mm, < 1/4 inch) needles, inserted at 90° without raising a skin lift, resulted in SC tissue injections in more than 98% of cases. Insertion of 6-mm and 8-mm (> 1/4 inch) needles at 90° resulted in IM injections in 5% and 15% of cases, respectively, whereas insertion of 12.7-mm (1/2 inch) needles at 90° or 45° resulted in IM injections in 45% and 21% of cases, respectively (Gibney et al., 2010). The survey findings show that respondents were injecting at 90° even when using longer needles. Use of a skin lift and a 45° angle is recommended for needles longer than 6 mm (1/4 inch; Figure 2F), whereas with short needles (4–6 mm), use of a skin lift and a 90° angle minimizes the risk of IM injection (Frid et al., 2010; Gibney et al., 2010; Akkus et al., 2012).

The survey found that approximately half of respondents were using the air-bubble technique and half were using the air-purge technique, with many of the latter indicating that this was based on how they had been taught or on concerns about injecting air. However, the air-bubble technique has demonstrated reduced injection-site bruising and pain and increased patient satisfaction (Kurtin et al., 2012; Moore et al., 2007; Wooldridge & Jackson, 1988).

Bortezomib is considered to be an irritant, and therefore it is reasonable to change needles after drawing up the medication and prior to injection, to avoid leaving an injection track (Kurtin et al., 2012). Of note, a randomized study of 100 patients demonstrated that changing the needle prior to administering IM injections reduced injection-site pain (Agac & Gunes, 2011).

Although purging the needle of air prior to SC or IM injection has been traditionally recommended and is a frequently taught technique (Hunter, 2008; McConnell, 1990; Pope, 2002; Rushing, 2004), two studies have demonstrated that using a dry needle and adding a 0.1 mL air bubble in the syringe, compared with purging the needle, resulted in a significant decrease in site redness (*p* = .001) and improved patient satisfaction and adherence to therapy (Moore et al., 2007; Wooldridge & Jackson, 1988). It is important to note that the air-bubble technique is not appropriate for IV injections.

The duration of injection varied between the respondents to this survey, although SC bortezomib was primarily being administered at the rate recommended for IV bortezomib: 3 to 5 seconds (Millennium Pharmaceuticals Inc., 2014) or slightly longer. However, studies have demonstrated that SC injections of 30 seconds, or 10-second injections followed by a 10-second delay before withdrawing the needle, resulted in significantly less bruising and pain compared with 10-second injections alone (Balci Akpinar & Celebioglu, 2008; Zaybak & Khorshid, 2008).

The majority of respondents agreed that consistency in the administration technique for SC bortezomib was important, and all nurses agreed that the development of a practice guideline would be important for standardizing SC administration of bortezomib. Notably, the survey findings were consistent in a number of aspects with the findings of the International Myeloma Foundation’s Nurse Leadership Board Roundtable Meeting regarding SC administration of bortezomib (International Myeloma Foundation, 2013). However, it is interesting that there was no association or correlation between whether a clinic had a standard guideline and the use of specific injection techniques. These findings are consistent with the clinical literature, which indicates that although guidelines may be in place, clinicians’ (nurses’ and physicians’) adherence and knowledge of them are inconsistent, even when clinicians agree about the importance of following them (Binner, Ross, & Browner, 2011; Côté, Gagnon, Houme, Abdeljelil, & Gagnon, 2012; Martin & Larson, 2003; Nirenberg, Reame, Cato, & Larson, 2010; O’Boyle, Henly, & Larson, 2001; Waters, Corrigan, Gatesman, & Smith, 2013).

## IMPLICATIONS FOR ADVANCED PRACTICE

Half of oncology nurses responding to the survey indicated APRNs in their practice share responsibility for ordering SC bortezomib, thus playing an integral role in directing patient care, identifying and addressing clinical problems, and maximizing effective treatment outcomes.

The survey suggests nurses may be using different SC injection techniques, which may contribute to injection-site irritation. Therefore, APRNs may consider evaluating SC injection techniques being used in practice settings to promote consistent clinical practice and professional evidence-based collaboration.

The findings of this survey can help inform the development of practice guidelines for the SC administration of bortezomib, with the aim of achieving greater consistency among oncology nurses and improving patient outcomes. Guidelines would ideally include preparation of bortezomib for SC injection; anatomic sites appropriate for SC injections, including injection site rotations; changing needles prior to injections; use of an air bubble; needle length, skin lift, and angle of insertion; and injection duration. Evidence supporting the recommended techniques and graphics when possible, particularly for site rotation, the air-bubble technique, skin lift, and angle of inserting needles, should also be included.
